# Enhanced Photoelectrochemical Properties of Ti^3+^ Self-Doped Branched TiO_2_ Nanorod Arrays with Visible Light Absorption

**DOI:** 10.3390/ma11101791

**Published:** 2018-09-20

**Authors:** Jingyang Wang, Xiantao Wang, Jun Yan, Qi Tan, Guijie Liang, Shaohua Qu, Zhicheng Zhong

**Affiliations:** 1 School of Physics and Electronic Engineering, Hubei University of Arts and Science, Xiangyang 441053, China; 1502060441@gmail.com (X.W.); 18827551096yj@gmail.com (J.Y.); tanqi9504@gmail.com (Q.T.); qushaohua@hbuas.edu.cn (S.Q.); 2 Hubei Key Laboratory of Low Dimensional Optoelectronic Materials and Devices, School of Physics and Electronic Engineering, Hubei University of Arts and Science, Xiangyang 441053, China; liangguijie@hbuas.edu.cn (G.L.); zhongzhicheng@hbuas.edu.cn (Z.Z.)

**Keywords:** branched TiO_2_ nanorod arrays, Ti^3+^ self-doped, hydrothermal, magnetron sputtering, photoelectrochemical properties

## Abstract

A novel Ti^3+^ self-doped branched rutile TiO_2_ nanorod arrays (NRAs) was successfully grown on an F-doped tin oxide (FTO) transparent conductive glass by a combined hydrothermal and magnetron sputtering method. Surface morphology, structure, optical properties, and photoelectrochemical behavior of the branched TiO_2_ NRAs are determined. Using TiO_2_ nanoparticles (NPs) deposited on the top of the nanorods as seeds, TiO_2_ nanobranches can easily grow on the top of the nanorods. Moreover, the Ti^3+^ defects in the TiO_2_ NPs and associated oxygen vacancies, and the nanobranches expend the optical absorption edge of the TiO_2_ NRAs from 400 nm to 510 nm. Branched TiO_2_ NRAs exhibit excellent photoelectrochemical properties compared to the pure TiO_2_ NRAs, as revealed by photoelectrochemical measurements. This enhanced photoelectrochemical properties is induced by the increased surface area and expanded optical absorption range. Due to their favorable characteristics, these novel branched TiO_2_ NRAs will provide a new path to the fabrication of hierarchical nanostructured materials.

## 1. Introduction

In the past few years, titanium oxide (TiO_2_) nanoarrays (i.e., nanotube, nanorod, and nanowire (NW) arrays) have attracted considerable attention as photoelectrodes in various photoelectrochemical (PEC) applications [1,2,3]. Compared with conventional TiO_2_ nanoparticle (NP)-film photoelectrodes, TiO_2_ nanoarray photoelectrodes have direct and ordered carrier transport channels, which can decouple a minor amount of charge diffusion paths into different directions to improve charge collection efficiency [4,5]. Moreover, with vertically aligned one-dimensional (1D) structures, light scattering and absorption can be improved greatly [6].

Among these 1D TiO_2_ nanoarrays, TiO_2_ nanorod arrays (NRAs) have been recognized as one of the most anticipated TiO_2_ nanoarrays due to unique physical and chemical properties and excellent stability [7,8]. Nevertheless, TiO_2_ NRAs are limited to a small specific surface area and a wide band gap (3.2 eV). Many efforts have been made to overcome these limitations of TiO_2_ NRAs. The growth of branched TiO_2_ NRAs has been proven to be an effective way to increase the specific surface area [9,10,11,12]. Wang et al. [9] prepared branched rutile TiO_2_ NRAs via a two-step wet chemical synthesis process, and Cho and co-workers [10] also prepared a kind of branched TiO_2_ NRAs by a two-step hydrothermal process by using a TiCl_3_ aqueous solution as a precursor for the growth of branches. Similarly, flower-like branched TiO_2_ NRAs have been prepared by Liu and co-workers with a modified two-step hydrothermal method [12]. It is found that the anatase/rutile junctions on the surface of TiO_2_ nanorod are favorable to the photoelectric properties of NRAs. Nevertheless, these TiO_2_ branches are still relatively short. Besides, many attempts such as element doping [13] and sensitization with dyes or narrow band-gap semiconductors [14,15] have been made to extend optical absorption ranges of TiO_2_ NRAs. Unfortunately, the stability of these dyes and semiconductors is not satisfactory. Thus, at the present, it is still attractive to develop novel branched TiO_2_ NRAs that exhibit a larger surface area and a wider absorption range at the same time. To the best of our knowledge, such attempts have been rarely reported.

In this paper, following our previous work on synthesis of branched TiO_2_ NRAs and TiO_2_ NP/NRA composites [16,17], a combined magnetron sputtering and hydrothermal method has been developed to grow Ti^3+^ self-doped branched TiO_2_ NRAs. With the larger surface area and improved optical absorption, the PEC properties of branched TiO_2_ NRAs are significantly improved compared with those of pure TiO_2_ NRAs.

## 2. Materials and Methods

First, TiO_2_ NRAs were prepared on TiO_2_-seeded FTO transparent conductive glass using the typical hydrothermal method [17]. Half a milliliter titanium butoxide was added to a 24-mL de-ionized (DI) water and hydrochloric acid (mass fraction: 36.5–38%)-mixed solution (a volume ratio of DI water and hydrochloric acid is 1:1). The mixture was stirred for 10 minutes and transferred to a 50-mL Teflon lined stainless steel autoclave. A TiO_2_-seeded FTO transparent conductive glass was put in the Teflon liner and heated to 150 °C for 5 h. After the growth of TiO_2_ NRAs, Ti NPs were deposited on the top of the TiO_2_ NRAs by direct current (DC) magnetron sputtering in a physical vapor deposition system (PVD75, Kurt J. Lesker Company, Jefferson Hills, PA, USA). A high-purity titanium wafer (99.995%, ZhongNuo Advanced Material Technology CO., LTD, Beijing, China) was used as a sputtering target. The base vacuum of the sputtering chamber was 1.0 × 10^−6^ Torr and the deposition pressure was carried out at 8 m Torr by using Ar gas (99.999%) as the working gas. The source-to-sample distance and the sample rotation speed were 150 mm and 6 rev·min^−1^, respectively. The sputtering power was 100 W and maintained for 60 min. The substrate temperature was kept at room temperature. The prepared products were annealed in air at 450 °C for 1 h to form TiO_2_ NP/NRA composites.

For the formation of branched TiO_2_ NRAs, the prepared TiO_2_ NP/NRA composites were subjected to a second hydrothermal treatment. DI water (12.5 mL), HCl (12.5 mL), and titanium butoxide (0.15 mL) were used as the precursor. The mixture was added into the autoclave, to which the TiO_2_ NP/NRAs composite was placed in. The autoclave temperature was increased to 160 °C for 3 h. After the synthesis, the branched TiO_2_ NRAs were rinsed with DI water and ethanol. The final annealing was performed at 450 °C for 30 min.

The phase structures of as-prepared products were identified by X-ray diffraction (XRD, D8 Advance, Bruker, Madison, WI, USA) with Cu-Kα radiation (λ = 1.54060 Å), and the 2*θ* scanning speed was 5°/min. The morphologies and microstructure were studied on a field-emission scanning electron microscope (FESEM, Hitachi, S-4800 and acceleration voltage was 10 kV, Tokyo, Japan). Transmission electron microscopy (TEM) and high-resolution TEM (HRTEM) images were obtained on an FEI Tecnai G2 F30 microscope operating at 200 KV, Hillsboro, OR, USA. X-ray photoelectron spectroscopy (XPS) was conducted on an ESCALAB 250Xi (Thermo, Waltham, MA, USA) system with an Al-*Kα* X-ray source. The spot size was 500 μm and the energy step size was 0.1 eV. Diffuse reflectance and absorption spectra were measured using a UV-Vis spectrophotometer (UV-3600, Shimadzu, Kyoto, Japan) equipped with integrating spheres with a scanning range of 300 nm to 700 nm by scanning at a high scan speed. The sample interval and slit width were 0.5 nm and 20 nm, respectively. PEC measurements and electrochemical impedance spectroscopy (EIS) were determined on an electrochemical workstation (Autolab/PGSTAT302N, Metrohm Autolab, Herisau; Switzerland) with a standard three-electrode electrochemical cell in a 0.5-M Na_2_SO_4_ solution. TiO_2_ NRAs, or branched TiO_2_ NRAs were used as working electrodes; a platinum plate electrode (dimension: 15 mm × 15 mm) was used as the counter electrode and Ag/AgCl in saturated KCl as the reference electrode. A Xe lamp with an intensity of 100 W/cm^2^ was used as the illumination source. The active area of the working electrode was 1.5 cm^2^. The frequency range of EIS measurements was from 0.01 Hz to 100 kHz and the ac amplitude was set at 10 mV.

## 3. Results and Discussion

**Figure 1:**
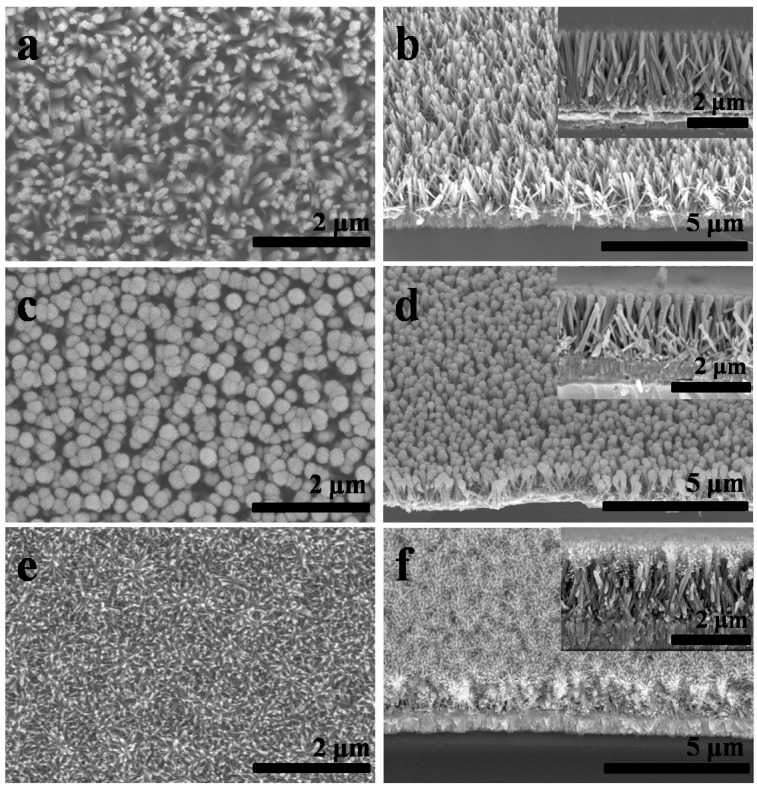
Surface and side-view SEM images of the TiO_2_ NRAs (**a**,**b**); NPs/NRAs (**c**,**d**); and branched NRAs (**e**,**f**). The insets in b, d and f show high-magnification SEM images of TiO_2_ NRAs, NPs/NRAs and branched NRAs, respectively.

The morphologies of TiO_2_ NRAs, TiO_2_ NP/NRA composites, and branched TiO_2_ NRAs are shown in Figure 1a–f. Figure 1a displays the surface SEM image of TiO_2_ NRAs, exhibiting a unified rod-like structure. Figure 1b presents the side-view SEM image of the same sample, and its inset shows higher-magnification image of the arrays, exhibiting that the nanorods grew nearly vertically on the substrate with a length of about 2.5 μm. When Ti NPs were deposited on the top of TiO_2_ NRAs and annealed to form TiO_2_ NPs/NRAs composites, the initial square morphology of the nanorods changed to sphere morphology, as shown in Figure 1c,d. The match-like TiO_2_ NP/NRA composites were then subjected to the second hydrothermal growth, and tree-like branched TiO_2_ NRAs were formed successfully (see Figure 1e,f). It was noticed that the nanobranches densely and uniformly covered TiO_2_ nanorods on the top. The length of nanobranches was much longer than those previously reported, which are grown on the side surface of TiO_2_ nanorods [10,11,12]. Close observation (the inset in Figure 1f) shows that the branches mainly grew on the top of the nanorods and diverged in all directions to form a spherical shape. Obviously, these nanobranches significantly increased the surface area of the TiO_2_ NRAs.

**Figure 2:**
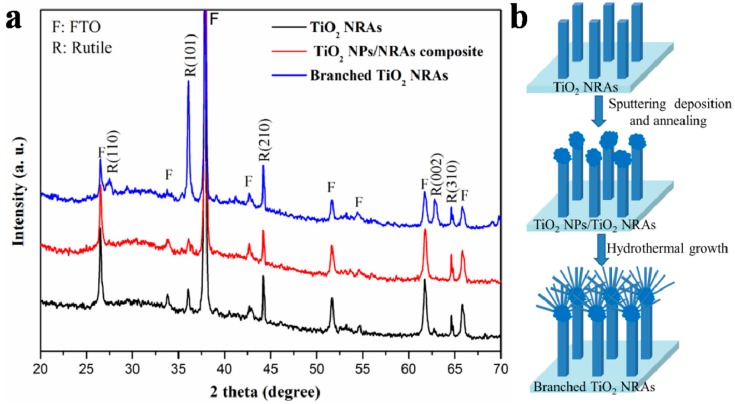
(**a**) XRD patterns of TiO_2_ NRAs, Nps/NRAs and branched NRAs; and (**b**) schematic growth of branched TiO_2_ NRAs.

Figure 2a shows the XRD patterns of TiO_2_ NRAs, TiO_2_ NP/NRA composites, and branched TiO_2_ NRAs. The XRD patterns showed that all the crystal structures of these three samples could be classified as the tetragonal rutile phase of TiO_2_. The peak intensities of the (101), (110), and (002) planes of branched TiO_2_ NRAs were stronger than those of pure NRAs. This indicated that the branches were well crystallized, and the growth mechanism is the same as for the TiO_2_ nanorod trunk. The growth rate on the (101) plane of rutile TiO_2_ nanorods is faster than that on the (110) plane [7,18], explaining the greatly enhanced intensity of the diffraction peak of the (101) plane with respect to the other diffraction peaks. Figure 2b shows the formation process of the branched TiO_2_ NRA structure. The deposited TiO_2_ NPs at the top of the nanorods served as crystal seeds for subsequent branching growth at energetically favorable sites on the top of nanorods. As shown in Figure 1f, TiO_2_ seeds were grown into dendritic branches, while the nanorod trunks did not grow further.

**Figure 3:**
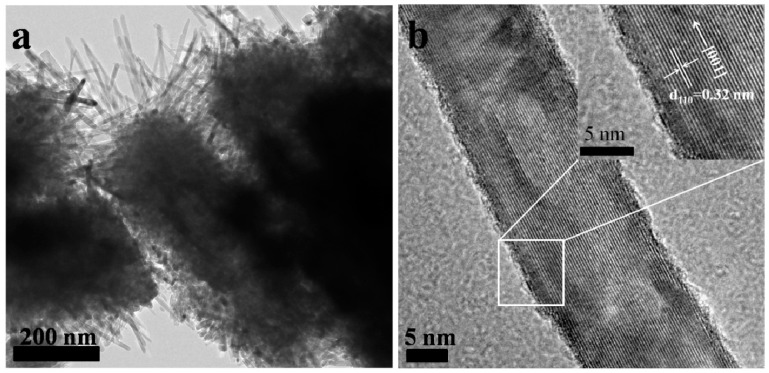
Overlapping of TEM and HRTEM images of (**a**) branched TiO_2_ NRAs and (**b**) a single branch.

Figure 3a displays the TEM image of branched TiO_2_ NRAs. It can be seen that nanobranches with about 200 nm in length and 40 nm in diameter uniformly covered nanorods on the top. The HRTEM image of a single branch is shown in Figure 3b, which exhibited clear and discernible lattice fringes, indicating good crystallinity of TiO_2_ nanobranches. The lattice constant with an interplanar spacing of 0.32 nm in the parallel direction to the length suggested the nanobranches were also crystallized to tetragonal rutile phase and had the same [001] growth direction as nanorods.

**Figure 4:**
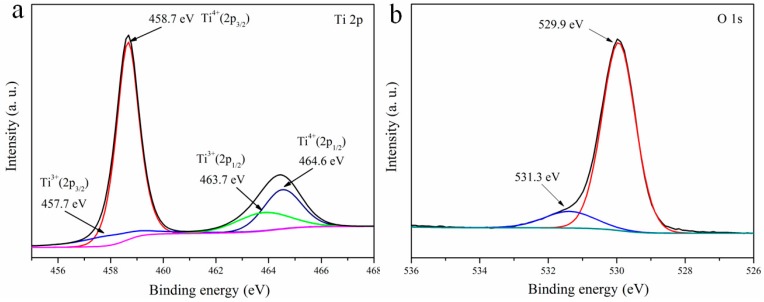
XPS spectra of the branched TiO_2_ NRAs: (**a**) Ti 2p; (**b**) O 1s.

XPS was exploited to characterize the chemical valence state and composition of branched TiO_2_ NRAs. As shown in Figure 4a, the observed two peaks at 458.6 and 464.4 eV corresponded to Ti 2p_3/2_ and Ti 2p_1/2_ of the branched TiO_2_ NRAs, respectively. Two Ti 2p peaks can be deconvoluted into four peaks, including the peaks of Ti^3+^ 2p_3/2_ at 457.7 eV and Ti^3+^ 2p_1/2_ at 463.7 eV, which indicates the existence of Ti^3+^ species [19]. These Ti^3+^ species are introduced by TiO_2_ NPs at the top of the nanorods [20,21,22,23]. In this case, the Ti NPs were deposited on the top of the nanorods by magnetron sputtering, and then annealed in air to form TiO_2_ NPs, which can also form a certain amount of reduced TiO_2_ (TiO_2−x_) and result in the formation of Ti^3+^ species. On the other hand, in order to maintain the charge equilibrium, oxygen vacancies were formed around Ti^3+^ defects. The O 1s spectrum is shown in Figure 4b. The main peak at 529.9 eV can be assigned to the O lattice of TiO_2_ and the binding energy of 531.3 eV can be ascribed to lattice oxygen (Ti–O) and oxygen in surface –OH groups [24]. Ti^3+^ defects and oxygen vacancies existing in the branched TiO_2_ NRAs can cause the formation of TiO_2_-localized states, thus promoting the separation of photoinduced electrons and holes [25,26].

**Figure 5:**
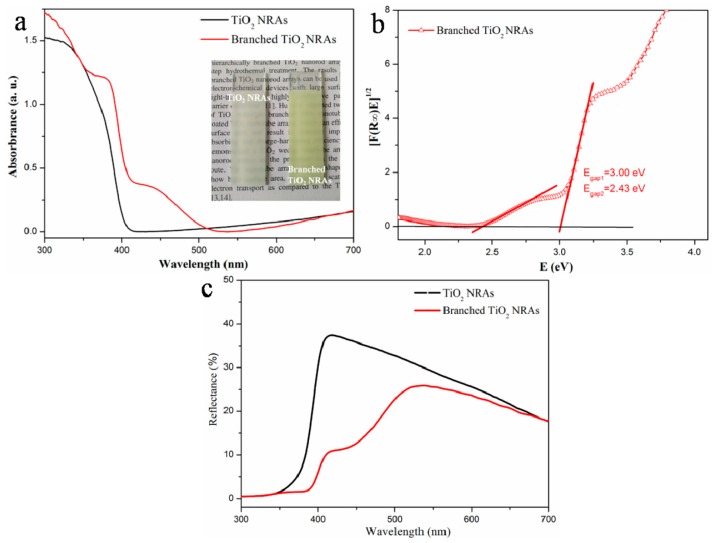
(**a**) Diffuse reflection absorption spectra, with an inset displaying the photo images of TiO_2_ NRAs and branched NRAs; (**b**) transformed diffuse reflection absorption spectra of the branched NRAs and (**c**) diffuse reflection spectra of the TiO_2_ NRAs and branched NRAs.

The light absorption properties of branched TiO_2_ NRAs and pure TiO_2_ NRAs were studied by diffuse reflection absorption spectroscopy. The rutile TiO_2_ NRAs exhibited a single absorption edge at 400 nm, consisting with the rutile TiO_2_ band gap of 3.0 eV. Distinct from the TiO_2_ NRAs, the spectrum of the branched TiO_2_ NRAs (Figure 5a) exhibited a structure of multiple band gaps, and a new absorption edge appeared around the 510 nm, which was a strong indicator for the unique geometric structure. The inset in Figure 5a shows the photographic images of TiO_2_ NRAs and branched TiO_2_ NRAs. The pure TiO_2_ NRAs showed gray white color, while the branched NRAs changed to light yellow. Furthermore, a plot of the modified Kubelka–Munk function *[F(R∞)E]*^1/2^ vs. the energy of absorbed light *E* was used to calculate values of *E*_gap1_ and *E*_gap2_ to be 3.0 eV and 2.43 eV for these two band gaps, respectively, as shown in Figure 5b. The multiple band gaps presented in the branched NRAs should result from two reasons: (i) defect energy levels introduced by Ti^3+^ species in the reduced TiO_2_ NPs at the top of the nanorods, which was confirmed by the XPS results. It has been demonstrated that reduced TiO_2_ (TiO_2−x_), which contains the Ti^3+^ or oxygen vacancy, exhibit visible light absorption [23,24,25]; (ii) the quantum confinement of the electrons in the TiO_2_ nanobranches. Previous studies have demonstrated that, when the diameter of anatase TiO_2_ NWs reduces to 40 nm, the multiple band-edge absorptions could occur, which can be induced by quantum confinement [27,28]. In this work, the morphology of TiO_2_ nanobranches was similar to that of TiO_2_ NWs, and the diameter of nanobranch was about 40 nm. Therefore, the absorption step could be also attributed to the quantum confinement in rutile TiO_2_ nanobranches. Figure 5c presents the reflectance spectrum of the TiO_2_ NRAs and branched NRAs. Obviously, branched TiO_2_ NRAs exhibited lower reflectance as compared to the pure TiO_2_ NRAs. The branched nanorod structure with higher surface roughness can increase the incident light scattering path, and result in the reflectivity reduction of branched NRAs [29,30]. However, it was also noticed that the branched TiO_2_ NRAs had lower absorption than that of TiO_2_ NRAs at the wavelength range from 510 nm to 700 nm even though the surface area increased, which can be ascribed to the light scattering effect from the increased surface roughness of the branched geometric structure [12,31,32].

**Figure 6:**
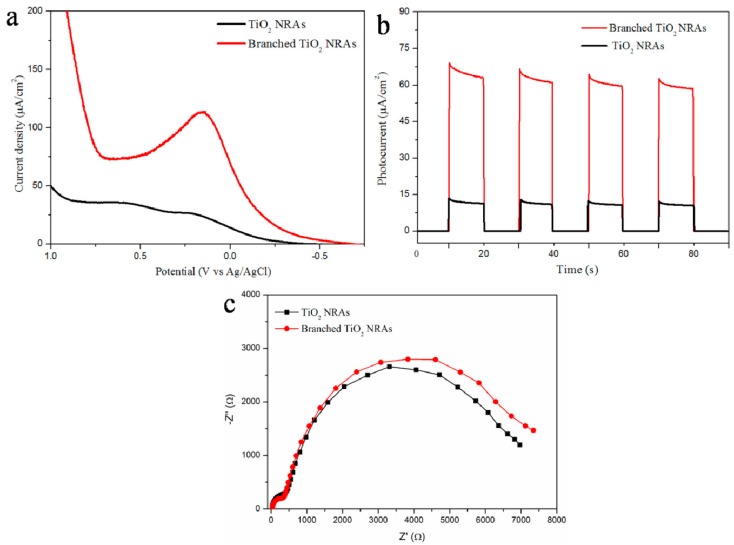
(**a**) Current-density versus voltage (*J*-*V*) curves and (**b**) photocurrent density response of TiO_2_ NRAs and branched NRAs; (**c**) Nyquist plots of TiO_2_ NRAs and branched NRAs based cells.

Figure 6a shows the linear sweep voltammpgrams curves of pure TiO_2_ NRAs and branched TiO_2_ NRAs under AM1.5G simulated sunlight. These results clearly showed that the photocurrent of branched TiO_2_ NRAs was much higher than that of the pure TiO_2_ NRAs film under visible-light illumination. The higher photocurrent indicated a higher efficiency in the separation of photon-generated electrons and holes, which resulted in a better PEC activity. Figure 6b shows the photocurrent response of TiO_2_ NRAs and branched NRAs under pulsed visible-light irradiation at zero bias. TiO_2_ NRAs and branched NRAs both exhibited the quick response to the switching of incident light, indicating a quick transfer of photogenerated electrons from the nanorod to the substrate [33]. This showed that the branched TiO_2_ NRAs had the same high electron transport efficiency as pure TiO_2_ NRAs. This conclusion was further confirmed by EIS spectroscopy (Figure 6c), as the exhibited large semicircle corresponds to the resistances of the TiO_2_/FTO and TiO_2_/electrolyte interfaces [34]. The diameter of the large semicircle measured for the cell using branched TiO_2_ NRAs as the photoanode was only slightly larger than that for the cell using pure TiO_2_ NRAs as the photoanode, suggesting that branched TiO_2_ NRAs still exhibit better electron transport properties and lower series resistances [35]. 

## 4. Conclusions

In summary, Ti^3+^ self-doped branched TiO_2_ NRAs with visible light absorption were successfully prepared by combining a hydrothermal method with magnetron sputtering technology. Using TiO_2_ NPs on the nanorods as seeds, the tree-like branched TiO_2_ NRAs can be easily formed. The Ti^3+^ defects and oxygen vacancies in TiO_2_ NPs and nanobranches expanded the absorption range of the TiO_2_ NRAs to visible light region. Based on the larger surface area, the expanded optical absorption range, and the better carrier transport properties, branched TiO_2_ NRAs exhibit better PEC activity than pure TiO_2_ NRAs, which makes them promising candidates for applications in PEC, photovoltaic, and photocatalytic devices.
